# The effect of the subthreshold oscillation induced by the neurons' resonance upon the electrical stimulation-dependent instability

**DOI:** 10.3389/fnins.2023.1178606

**Published:** 2023-05-09

**Authors:** Shoujun Yu, Wenji Yue, Tianruo Guo, Yonghong Liu, Yapeng Zhang, Sara Khademi, Tian Zhou, Zhen Xu, Bing Song, Tianzhun Wu, Fenglin Liu, Yanlong Tai, Xuefei Yu, Hao Wang

**Affiliations:** ^1^School of Biomedical Engineering, Southern Medical University, Guangzhou, China; ^2^Institute of Biomedical and Health Engineering, Shenzhen Institutes of Advanced Technology (SIAT), Chinese Academy of Sciences (CAS), Shenzhen, China; ^3^Graduate School of Biomedical Engineering, University of New South Wales, Sydney, NSW, Australia; ^4^School of Biomedical Engineering, Shanghai Jiao Tong University, Shanghai, China; ^5^Institute of Polymeric Materials, Sahand University of Technology, Tabriz, Iran; ^6^Key Laboratory of Health Bioinformatics, Chinese Academy of Sciences (CAS), Shenzhen, China

**Keywords:** threshold fluctuation, neural modeling, Circuit-Probability theory, neural oscillation, subthreshold oscillation

## Abstract

Repetitive electrical nerve stimulation can induce a long-lasting perturbation of the axon's membrane potential, resulting in unstable stimulus-response relationships. Despite being observed in electrophysiology, the precise mechanism underlying electrical stimulation-dependent (ES-dependent) instability is still an open question. This study proposes a model to reveal a facet of this problem: how threshold fluctuation affects electrical nerve stimulations. This study proposes a new method based on a Circuit-Probability theory (C-P theory) to reveal the interlinkages between the subthreshold oscillation induced by neurons' resonance and ES-dependent instability of neural response. Supported by *in-vivo* studies, this new model predicts several key characteristics of ES-dependent instability and proposes a stimulation method to minimize the instability. This model provides a powerful tool to improve our understanding of the interaction between the external electric field and the complexity of the biophysical characteristics of axons.

## 1. Introduction

Function electrical stimulation assists purposeful movement by increasing the plasticity for motor function. By applying electrical stimulation to the paralyzed muscles, electrical nerve stimulation makes the coordinated muscle contract in a sequence that allows paralyzed patients to perform tasks such as standing, walking, or grasping a key (Rushton, 1997; Benabid, 2003; Peckham and Knutson, 2005; Dayan and Cohen, 2011; Sabbah et al., 2011; Famm et al., 2013; Marquez-Chin and Popovic, 2020). However, more detailed neurological mechanisms underlying electrical stimulation are still unclear. For example, the electrical stimulation-dependent (ES-dependent) instability of neural response is a well-known phenomenon. The axon's excitability has a non-monotonic fluctuation with repetitive stimulation. This unstable excitability was observed from single-cell-based patch-clamping electrophysiology (Bostock and Grafe, 1985) and muscular response to functional electrical stimulation (FES) (Potts et al., 1994; Bostock et al., 2005; Moldovan and Krarup, 2006). It is widely believed that the origin of this instability is the fluctuation of the axon's threshold voltage. Thus, this phenomenon is also called “threshold fluctuation” or “excitability fluctuation” in previous studies (Ten Hoopen et al., 1963; Potts et al., 1994; Kiernan et al., 1996; Bostock et al., 2005; Moldovan and Krarup, 2006).

Since this instability will affect the FES performance of the clinical treatment of diseases by electrical nerve stimulations, it concerned the researchers in this area. Many experimental studies have been conducted to investigate more detailed biological mechanisms in order to minimize the unstable effect (Ten Hoopen et al., 1963; Bostock and Grafe, 1985; Potts et al., 1994; Kiernan et al., 1996; Chen et al., 1999; Shefner, 2001; Moldovan and Krarup, 2004, 2006; Bostock et al., 2005; Krishnan and Kiernan, 2006; Krishnan et al., 2006; George and Bostock, 2007; Boërio et al., 2009, 2011; Burke et al., 2009; Baumann et al., 2010; Trevillion et al., 2010; Bucher and Goaillard, 2011; Sittl et al., 2011; Kudina and Andreeva, 2014, 2017; Urriza et al., 2016; Jankowska et al., 2017; Hageman et al., 2018; Kaczmarek and Jankowska, 2018; Sleutjes et al., 2018; Jankowska and Hammar, 2021; Deletis et al., 2022). Major experimental observations and concluded principles of threshold fluctuation are summarized below.

The threshold fluctuation is caused by the perturbation of the membrane potential of the axon, where the induced membrane potential perturbation can last more than 100 ms, longer than the refractory period of the axonal action potential (Adrian and Lucas, 1912; Gasser and Grundfest, 1936; Gilliatt and Willison, 1963; Raymond, 1979; Potts and Young, 1981; Barrett and Barrett, 1982; Stys and Ashby, 1990). Thus, it can be excluded that the neural refractory period is the major factor contributing to the ES-dependent instability (Potts et al., 1994).

The observed membrane potential perturbation happens in the vicinity of the stimulating electrode (Bostock et al., 2005). Therefore, although the EMG (Electromyography) signal is involved in evaluating the change of excitability for most relevant experiments, the muscle activation is irrelevant to the instability (Potts et al., 1994; Bostock et al., 2005; Moldovan and Krarup, 2006).

Subthreshold stimulations can also induce threshold fluctuation. Thus, the membrane potential perturbation happens as long as local electrical stimulations are applied, whether the action potential fires or not (Potts et al., 1994; Bostock et al., 2005; Moldovan and Krarup, 2006).

The relationship between the electrical stimulation parameter and the change in excitability is definitive (Bostock and Grafe, 1985; Potts et al., 1994; Stys and Waxman, 1994; Kiernan et al., 1996; Chen et al., 1999; Moldovan and Krarup, 2004, 2006; Bostock et al., 2005; Krishnan et al., 2006; George and Bostock, 2007; Boërio et al., 2009, 2011; Burke et al., 2009; Trevillion et al., 2010; Bucher and Goaillard, 2011; Sittl et al., 2011; Hageman et al., 2018; Sleutjes et al., 2018; Jankowska and Hammar, 2021). Previous studies assumed noise as the origin of threshold fluctuation (Ten Hoopen and Verveen, 1963; Lecar and Nossal, 1971), which contradicts the experimental observation.

However, the precise mechanisms underlying the observed ES-dependent instability remain unclear due to the lack of a proper theoretical model. In this study, we conducted both *in-vivo* and *in-silico* investigations to better understand this question. Our theoretical model based on a Circuit-Probability theory (C-P theory) (Wang et al., 2020) allows us to explore how the stimulation parameters affect the instability. It reveals that the subthreshold oscillation induced by neurons' resonance, a well-observed phenomenon in many studies (Jahnsen and Karnup, 1994; Puil et al., 1994; Gutfreund et al., 1995; Hutcheon et al., 1996), is the primary factor in determining ES-dependent instability. Meanwhile, our study provides a computational tool to characterize neurological mechanisms underlying electrical stimulation better.

## 2. Method

### 2.1. Animals preparation

Male Sprague-Dawley rats (~300 g) were used in experiments. Rats were housed and cared for in compliance with the guidelines of the Institutional Animal Care and Use Committee (IACUC) and were humanely euthanized after the experiment. The rat was placed in a transparent acrylic box and anesthetized with isoflurane (Iflurin, RingPu, China; R500-Series, RWD Life Science, China). Observe the paw retraction reflex and breathing rate to estimate the depth of anesthesia. After deep anesthesia, the rat was placed on a heating pad to maintain the body temperature at 37°C and worn an anesthesia mask during the experiment to ensure deep anesthesia. Remove the fur on the legs, disinfect the surgical area with 75% ethanol, and then expose and locate the sciatic nerve.

### 2.2. The electrical stimulation-dependent instability

**Figure 1:**
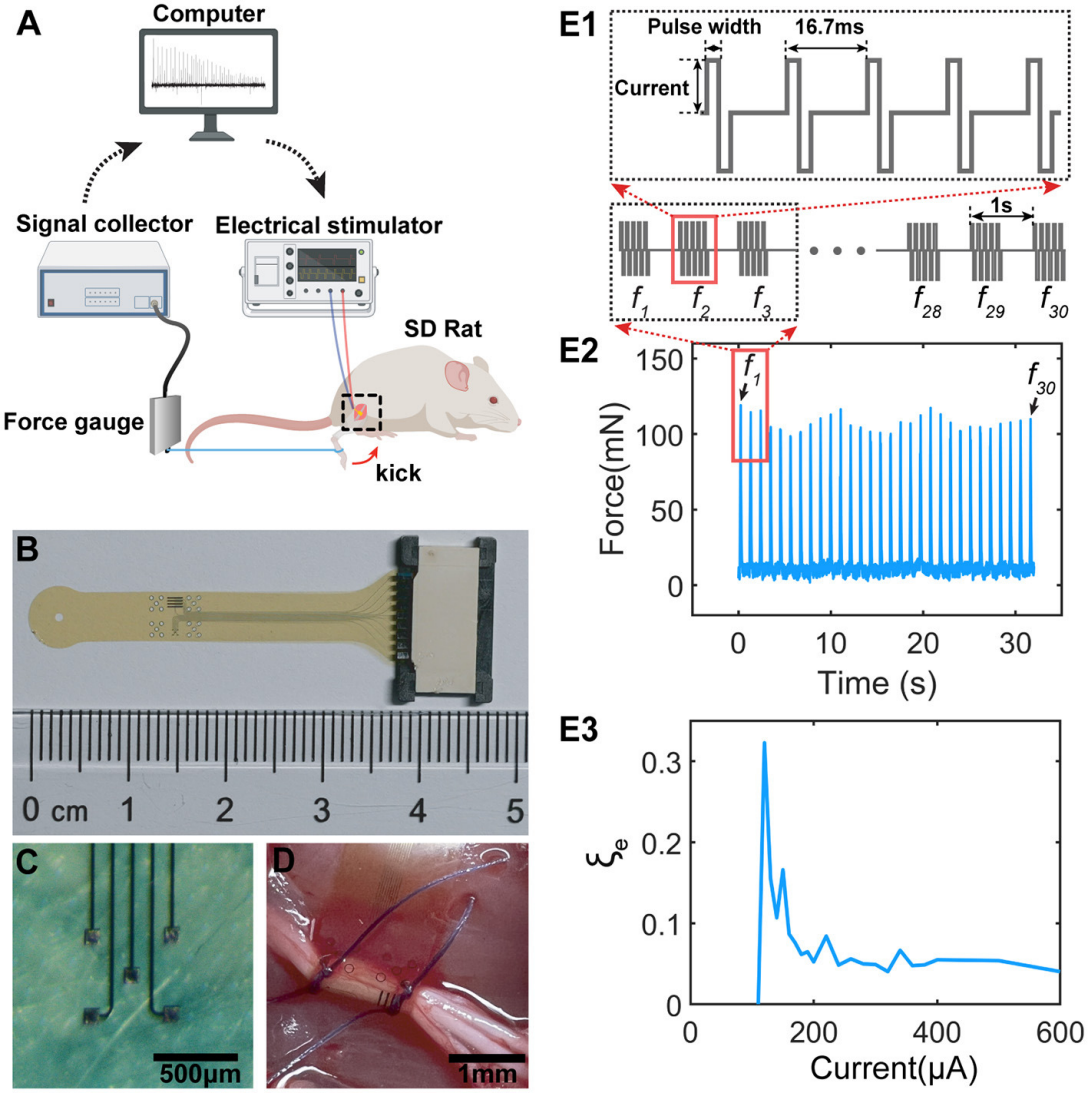
The testing setup, stimulation protocol and the result sample. **(A)** The testing setup for measuring the force generated by sciatic nerve stimulations; **(B)** The fabricated flexible neural probe used for sciatic nerve stimulations; **(C)** The electrode pads on the neural probe for stimulations; **(D)** The flexible neural probe implanted to the sciatic nerve; **(E1)** The stimulation protocol. Each stimulation train contains 5 current pulses with 16.7 ms as latency. The latency between each train is 1 s. **(E2)** The sample of measured force showing non-monotonous fluctuation. f_1_~f_30_ are the force pulses generated by the 30 pulse tains in **(E1)**. **(E3)** A sample of the instability curve of the measured force, defined as $\xi_{e}=\frac{F_{std}}{F_{mean}}$, by changing the current amplitudes.

We evaluated the instability by recording the patterns of kicking force of a rat's leg under sciatic nerve stimulation *in-vivo*. The experimental setup is shown in Figure 1A. A homemade flexible neural probe (Figure 1B) with five channels (Figure 1C) connected with an flexible printed circuit (FPC) connector was implanted on the sciatic nerve, shown in Figure 1C. The neural probe is of polyimide-Au-polyimide sandwiched structure fabricated by micro-electro-mechanical system (MEMS) technology. The detailed fabrication process is described in the previous study (Lee et al., 2017). Since two branches of the sciatic nerve, the tibial nerve and the common peroneal nerve, control the opposite kicking, one branch was cut in the downstream location to ensure a one-directional kicking. When an electrical stimulus (STG 4008, Multi-Channel Systems GmbH, Germany) was applied to the neural probe, a kicking force (forward or backward, depending on which branch was cut off) was recorded by the force gauge (ZL-X10 & ZL-620, Anhui Yaokun Biotechnology, China) connected to the rat's leg with a wire.

The stimulation protocol is shown in Figures 1E1–E3. Each stimulation train contains five pulses with 16.7 ms as the latency (Figure 1E1). Each train generates one force pulse with a period of 1 s. The detailed current waveforms, current amplitudes, and pulse widths will be provided for each test are shown in Table 1.

**Table 1:** Parameters of *in-vivo* experiments.

**No**	**Waveform**	**Pulse width (μs)**	**Current amplitude (μA)**
			
Fig1(E2)	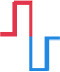	200	100
			
			
Fig1(E3)	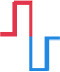	200	100:10:200, 200:20:380
			
			
Fig2(F)	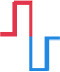	100,200,400	55:5:150
			
			
Fig4(C1)	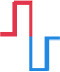	100	80:10:350
			
			
Fig4(C2)	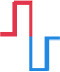	300	80:10:350
			
			
Fig4(C3)	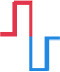	400	100:10:200,200:20:380
			
			
Fig5(B1)	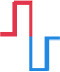	100:100:800	55:5:150
			
			
Fig9(A–C)red	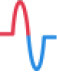	200	24:1:30, 30:2:50, 50:5:100, 100:20:160
			
			
Fig9(A—C)blue	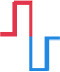	200	38:2:60, 60:5:80, 80:10:160
			
			
Fig10(B1&B2)	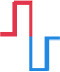	400	110:10:340
			
			
Fig10(C1&C3)	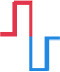	200	55:5:150
			
			
Fig10(C2)	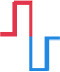	300	55:5:150
			
			
Fig12(A–C)	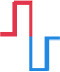	200	18

Figure 1E2 shows a typical sample of the measured force with specific testing parameters (Table 1-e2). The recorded force pulses show a non-monotonous fluctuation, indicating ES-dependent instability. The instability in the *in-vivo* experiment, ξ_*e*_, is defined as the standard deviation vs. the average value of the force amplitude:


(1) $$\begin{array}{l} \xi_{e}=\frac{F_{std}}{F_{mean}} \end{array}$$

An example of ξ_*e*_ curves is shown in Figure 1E3 by stimulating the nerve with a range of stimulus amplitudes. We identified a ES-dependent ξ_*e*_ pattern in terms of the ξ_*e*_ peak amplitude and position. This ES-dependent ξ_*e*_ is the major focus of this study.

### 2.3. Modeling instability by C-P theory

#### 2.3.1. A brief illustration of C-P theory

The major principle of the C-P theory can be explained by:

1. The electric field (E-field) across the axon membrane evokes action potentials. Since the phospholipid bilayer of a cell membrane can be modeled as a capacitor, this E-field is proportional to the cross-membrane potential, which is the voltage upon the capacitor of the cell membrane.
2. Since the input current and the generated voltage on the cell membrane do not share the same waveform, we need to build a circuit to calculate the voltage waveform.
3. There is a threshold voltage of nerve stimulation. Therefore, only the part of the voltage waveform exceeding the threshold has a probability of evoking the action potential.

**Figure 2:**
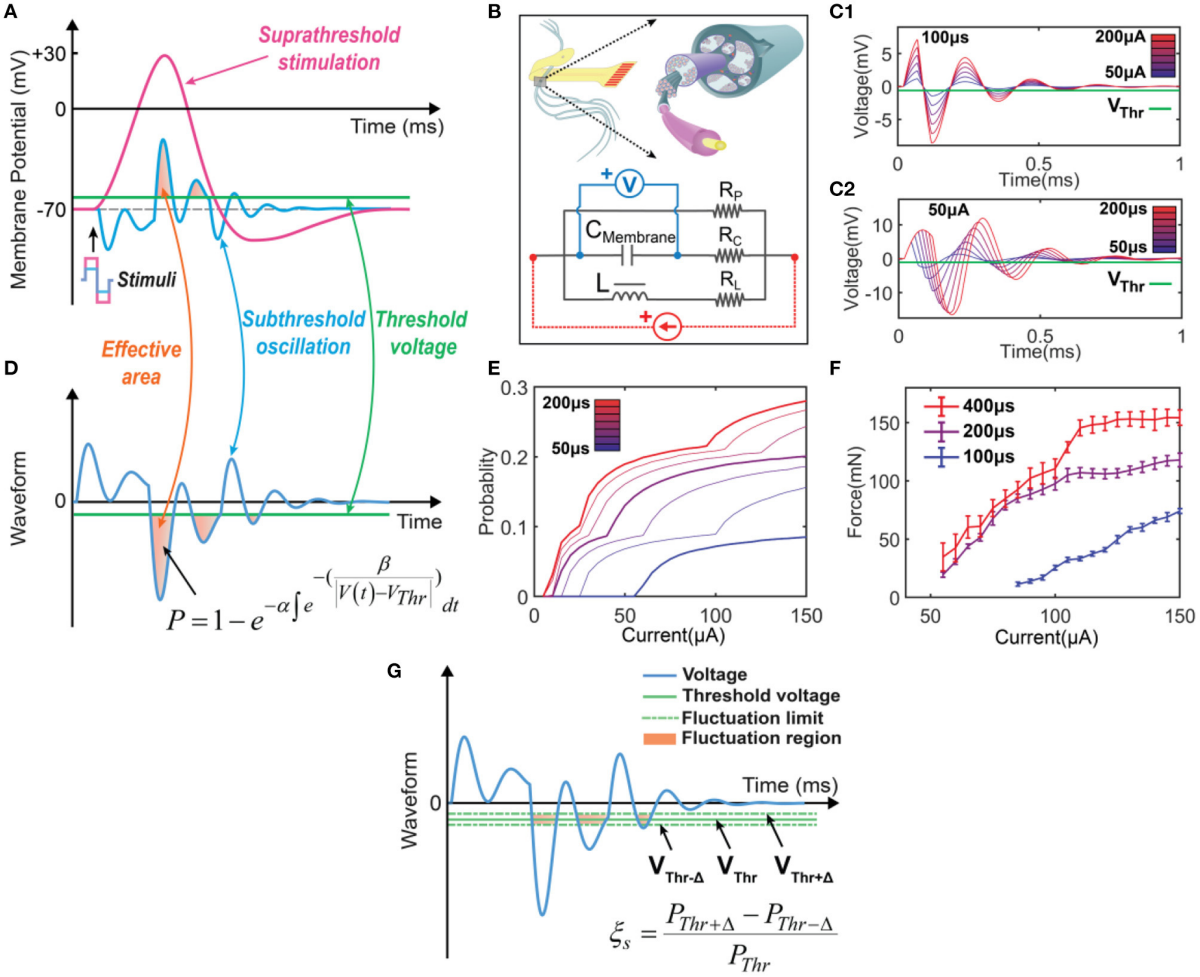
The concept of the C-P theory and the definition of instability in modeling. **(A)** The neural response of electrical stimulations: a suprathreshold stimulation will generate an action potential, while a subthreshold stimulation will induce a subthreshold oscillation. **(B)** The equivalent circuit with an RLC configuration to model the neurons. **(C)** The resultant voltage waveform across the *C*_*Membrane*_ was recorded when a positive-first biphasic square waveform current of varying amplitudes and pulse widths was applied. **(D)** The concept of C-P theory. The subthreshold oscillation can be duplicated by the voltage response of the RLC circuit in **(B)**. The part of the voltage exceeding the threshold will be involved in the calculation of the probability of generating an action potential. **(E)** Probability calculation of different current amplitudes by changing pulse widths (probability mapping). **(F)** Measured force curves of different current amplitudes by changing pulse widths in *in-vivo* testing. **(G)** The definition of ES-dependent instability: assuming that the threshold will have a fluctuation in the range of ±, the instability in modeling is defined as $\xi_{s}=\frac{P_{Thr+}-P_{Thr-}}{P_{Thr}}$.

Therefore, to calculate the probability proposed in Figure 2, we need two components. One is the equivalent circuit to duplicate the subthreshold oscillation. Another is the probability calculation based on the oscillating voltage. Our previous study demonstrated that this oscillating voltage could be duplicated by an RLC circuit shown in Figure 2B (Wang et al., 2020). In the circuit component, we build an RLC circuit to represent the passive property of neural tissue (Figure 2B). The capacitor *C*_*Membrane*_ represents the cell membrane. This circuit has an inductor *L*, which differs from the RC circuit used in conventional models such as the Hodgkin-Huxley model (H-H model) (Hodgkin and Huxley, 1952). We detailedly discussed the physical origins of the inductive element in the neural circuit (Wang et al., 2021). Generally, it has two origins. One is from the spiraling Schmidt-Lanterman incisure (SLI), the cytoplasmic channels in myelin sheaths. During the action potential, a spiraling current within SLI will generate a magnetic field, which functions similarly to a coil inductor. Thus, myelin can function as a real inductor. The primary evidence of the existence of this coil inductor is the non-random spiraling between adjacent myelin sheaths (Wang et al., 2021) and the explanation of Peter's quadrant mystery by the current in SLI (Liu et al., 2022), which are discussed in detail in our previous works. The second origin comes from the flexoelectricity of cell membrane, which is phenomenologically the same as piezoelectric effect (Petrov, 2006). Since the equivalent circuit to model the piezoelectric effect always follows a parallel RLC circuit, the flexoelectricity also contributes an equivalent inductor in the neural circuit. Thus, the coil inductor and the equivalent inductor are combined as one inductive element in the RLC circuit used in this study. *R*_*P*_ is the leakage resistance of the circuit. *R*_*C*_ and *R*_*L*_ are the resistors connected in series with the membrane capacitor and the inductor, respectively.

Figure 2D shows a typical subthreshold oscillation generated by the RLC circuit in Figure 2B. This voltage oscillation was reported in many biological experiments (Sjodin and Mullins, 1958; Araki et al., 1961; Freeman, 1961; Ranck, 1963; Guttman, 1969; Mauro et al., 1970; Scott, 1971; Takashima and Schwan, 1974; Hombl and Jenard, 1984; Koch, 1984; Hutcheon and Yarom, 2000; Dwyer et al., 2012; Mosgaard et al., 2015), particularly in the original study of the H-H model (Hodgkin and Huxley, 1952). When the threshold voltage is applied, the part of voltage exceeding the threshold will be involved in the equation of probability calculation:


$$\begin{array}{l} P=1-e^{-\alpha \int e^{-\left(\frac{\beta }{\left|V\left(t\right)-V_{Thr}\right|}\right)}dt} \end{array}$$

This equation is proposed and explained by our previous work on Circuit-Probability theory (Wang et al., 2020). α, β are parameters to be tuned to fit the experimental data. *V*_*Thr*_ is the threshold voltage, which is the difference between the resting potential and threshold voltage in Figure 2A. Due to the voltage oscillation, more than one block of the voltage waveform can be involved in the probability calculus. In the case shown in Figure 2D, three blocks are involved.

To better illustrate the C-P theory, we demonstrate how to derive and predict the results of *in-vivo* testing from modeling. The waveforms displayed in Figure 2C show the voltage oscillation across the *C*_*Membrane*_, generated by applying currents with varying amplitudes and pulse widths to the RLC circuit. As the current amplitude (Figure 2C1) and pulse width (Figure 2C2) increase, the effective areas for probability calculus also increase. Thus, we could obtain a specific probability mapping (Figure 2E), where thick curves can reproduce the measured force curves (Figure 2F) in *in-vivo* testing. Notably, due to the difficulty in accurately selecting the stimulation parameters during the testing process, the thin curves in the probability mapping (Figure 2E) cannot be obtained through *in-vivo* testing but can be simulated by C-P theory.

#### 2.3.2. Modeling the effect of threshold fluctuation

The model involved in this study is based on the C-P theory, which is briefly illustrated in Section 3.1. In this section, we introduce how to involved the threshold fluctuation in the model.

It is known that a successful electrical stimulation of an axon will activate an action potential (Figure 2A). However, if the stimulus is insufficient to evoke an action potential, an oscillating voltage waveform, which is normally called subthreshold oscillation, will be recorded in the experiment of patch-clamp (Figure 2A). This subthreshold oscillation is the voltage applied to the cell membrane. Thus, the part of the voltage higher than the threshold shall have a probability of evoking an action potential. It is emphasized that the subthreshold oscillation may not be really subthreshold. Part of the voltage still exceeds the threshold, providing a certain probability of activating an action potential. If the activation is failed, the recorded voltage is called subthreshold oscillation.

Since the *V*_*Thr*_ is the difference between the resting potential and the threshold voltage in Figure 2A, the fluctuation of the membrane potential, which is reported as the origin of the ES-dependent instability (Potts et al., 1994; Bostock et al., 2005; Moldovan and Krarup, 2006), can be modeled with a fluctuation of *V*_*Thr*_, as shown in Figure 2G. We assume that the threshold voltage can only fluctuate within a region defined by Δ, then the threshold voltage can be changed from the upper limit *V*_*Thr*+Δ_ to the lower limit *V*_*Thr*−Δ_, shown in Figure 2G. The instability in the simulation can be defined as


(2) $$\begin{array}{l} \xi_{s}=\frac{P_{Thr+\text{Δ}}-P_{Thr-\text{Δ}}}{P_{Thr}} \end{array}$$

where ξ_*s*_ refers to the instability in simulation. *P*_*Thr*_, *P*_*Thr*+Δ_ and *P*_*Thr*−Δ_ are the calculated probability by setting their own threshold voltages.

Now the threshold fluctuation is involved in the model and the definition of instability in modeling is obtained.

#### 2.3.3. Calculating the instability peaks in modeling

**Figure 3:**
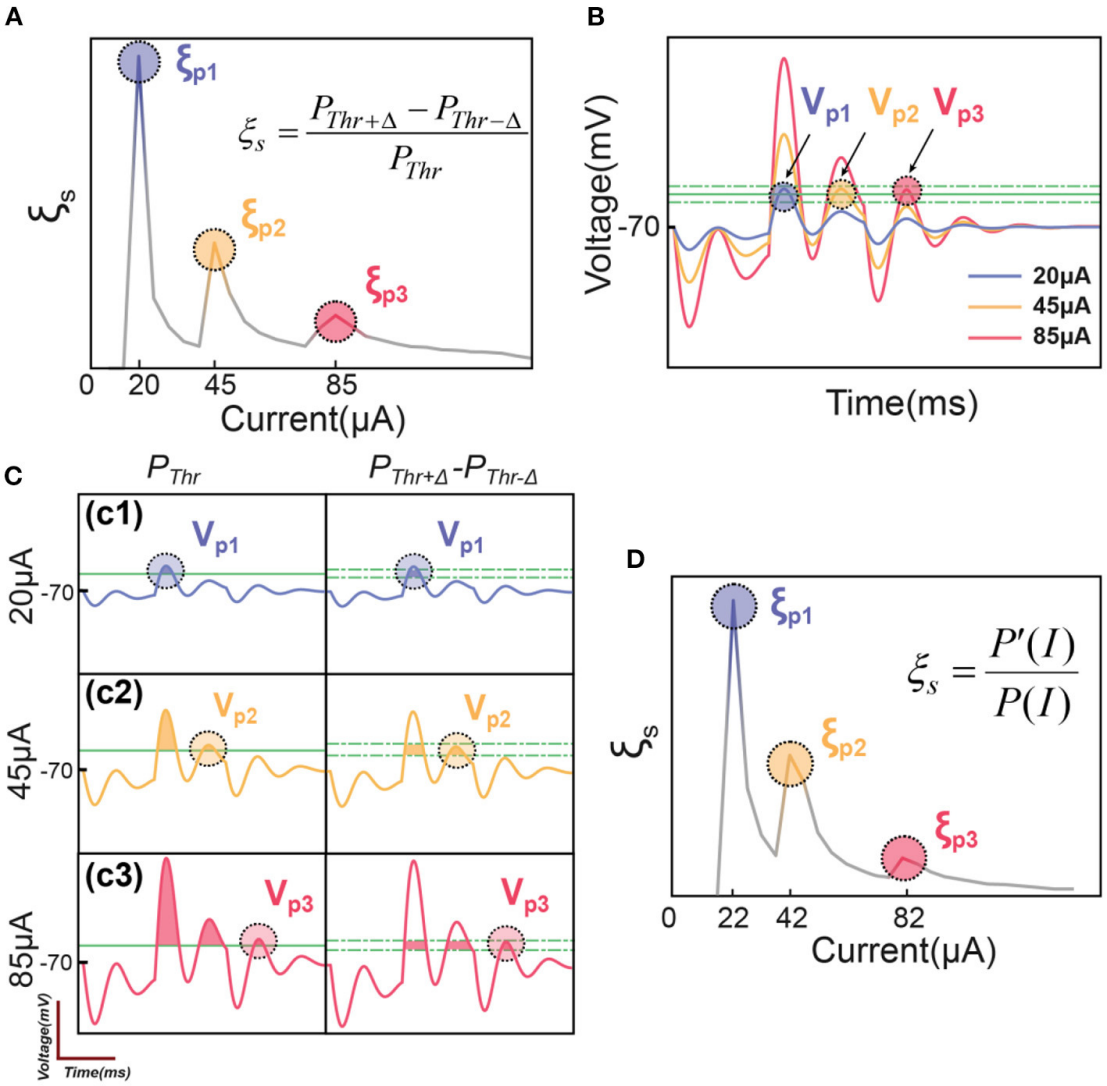
The illustrative explanation of the instability peaks in modeling. **(A)** Modeling results show several instability peaks in the instability curve by the definition of $\xi_{s}=\frac{P_{Thr+▵}-P_{Thr-▵}}{P_{Thr}}$; **(B)** The voltage oscillation shows several oscillation peaks corresponding to the instability peaks in **(A)**; **(C)** Illustrative explanation about how the voltage oscillation peaks induce instability peaks; **(D)** The modeling of instability peaks by the definition of $\xi_{s}=\frac{P^{\prime }\left(I\right)}{P\left(I\right)}$.

By using the C-P theory and the definition of instability in Figure 2, the instability peaks observed in testing (Figure 1E3) can be reproduced as shown in Figure 3.

A typical result of ξ_*s*_ by applying a positive-first biphasic square wave current with 400 μs pulse width is shown in Figure 3A. The curve has three peaks, reproducing the pattern shown in Figure 1E3. Here we need to explain why our model can reproduce the pattern of peaks in *in-vivo* tests.

For the representative voltage waveform shown in Figure 3B, three oscillation peaks can exceed the threshold, named as *V*_*p*1_, *V*_*p*2_ and *V*_*p*2_. Since these three voltage peaks are of different amplitudes, they reach the threshold in series by increasing the current. When the current is low, only *V*_*p*1_ reaches the threshold (Figure 3C1), the instability is


$$\begin{array}{l} \xi_{s}=\frac{P_{Thr+\text{Δ}}-P_{Thr-\text{Δ}}}{P_{Thr}}\approx \frac{P_{Thr}}{P_{Thr}}=100\% \end{array}$$

In this scenario, the instability reaches the maximum, shown as the first peak, ξ_*p*1_, in Figure 3A. Then the instability will decrease by increasing the current until the second voltage peak, *V*_*p*2_, reaches the threshold, shown in Figure 3C2. It will also induce a local maximum, ξ_*p*2_. But because the total probability, *P*_*Thr*_, on the denominator is higher, the amplitude of the second instability peak, ξ_*p*2_, is lower than ξ_*p*1_. Then by further increasing the current to make *V*_*p*3_ reach the threshold (Figure 3C3), a third instability peak, ξ_*p*3_, will appear, and its amplitude is lower than ξ_*p*2_. In summary, an instability peak will appear with a decreased amplitude whenever a new voltage peak exceeds the threshold.

The definition of instability, ξ_*s*_ in Figure 3A is based on the upper and lower limit of the threshold fluctuation, Δ. The amplitude of Δ will not change the qualitative results, which refer to the number and position of those peaks of ξ, but will determine the quantitative result, which refers to the height of the peaks. Here we will conduct a further derivation to make the definition of ξ free of Δ.

The fluctuation of the threshold changes the area of the voltage waveform involved in the probability calculus. Meanwhile, changing the amplitude of the input current can induce the equivalent effect. For example, increasing the threshold voltage is equivalent to reducing the current amplitude, while decreasing the threshold voltage is equivalent to increasing the current amplitude. Thus, equation (2) can be rewritten into another form:


(3) $$\begin{array}{l} \xi_{s}=\frac{P_{Thr+\text{Δ}}-P_{Thr-\text{Δ}}}{P_{Thr}}\to \xi_{s}=\frac{P\left(I+\text{Δ}I\right)-P\left(I-\text{Δ}I\right)}{P\left(I\right)} \end{array}$$

In this new equation (3), the fluctuation of the threshold is replaced by a change in the input current.

Since


$$\begin{array}{l} \underset{\text{Δ}I\to 0}{\lim}P\left(I+\text{Δ}I\right)-P\left(I-\text{Δ}I\right)=\frac{dP}{dI}\times 2\text{Δ}I \end{array}$$

Then further derive the equation (3) as follow:


(4) $$\begin{array}{l} \underset{\text{Δ}I\to 0}{\lim}\xi_{s}=\frac{dP}{dI}\times \frac{2\text{Δ}I}{P\left(I\right)} \end{array}$$

Since Δ*I* is always set as a constant, it can be neglected in the qualitative modeling; the equation (4) can be written as follow:


(5) $$\begin{array}{l} \xi_{s}=\frac{1}{P\left(I\right)}\times \frac{dP}{dI}=\frac{P^{\prime }\left(I\right)}{P\left(I\right)} \end{array}$$

This new definition does not rely on the value of Δ. The instability curve in Figure 3D modeling by equation (5) can generally reproduce the pattern in Figure 3A modeling by equation (2). But the width and the position of the peaks will have a slight shift, which is induced by reducing Δ to zero. In the following sections, all simulation results used equation (5) as the definition of ξ_*s*_.

## 3. Results

### 3.1. The number and position of peaks

**Figure 4:**
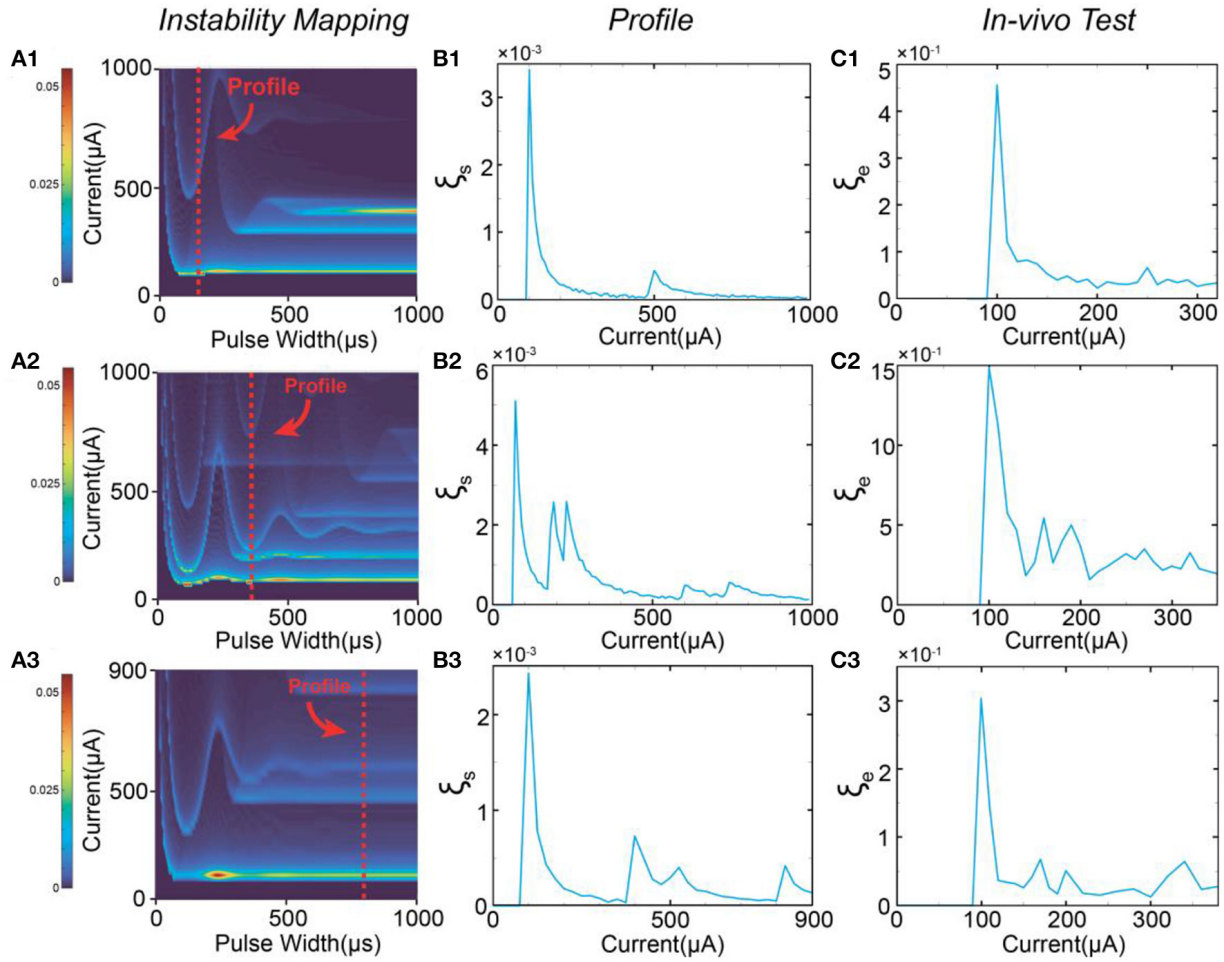
The patterns of the instability peaks in *in-vivo* tests are reproduced by modeling. **(A1–A3)** The heat map of the instability by changing both pulse width and current amplitude; **(B1–B3)** The captured profile of the instability curve at a specific pulse width; **(C1–C3)** The measured instability curves to be reproduced by **(B1–B3)**.

Based on the stimulus-response relationship given by the C-P model, we generated a full mapping of the instability by traversing all possible values of the current amplitude and the pulse width while keeping the same waveform. The simulation results will form a heat mapping shown in Figure 4A, called an instability mapping. All modeling settings and parameters are listed in Table 2. The result of the instability curve of a specific pulse width is just the profile of one cross-section of the instability mapping. In Figure 4A, the profiles captured at different pulse widths show different patterns (Figure 4B). This changing trend can also be observed in *in-vivo* testing (Figure 4C). Our model closely reproduces the observed patterns *in-vivo*. It should be noted that the current scales of instability curves in the *in-vivo* testing and simulation are different due to the omission of delta in the definition of instability and the uncertainty of threshold. Therefore, the comparison between Figures 4B, C shows that our simulation can only reproduce the relative scale of the curves and the scale of the peaks. Still, the quantitative results can not be accurately reproduced at present.

**Table 2:** Parameters of modeling.

**No**	**R_P_(kΩ)**	**R_C_(Ω)**	**R_L_(kΩ)**	**C(nF)**	**L(H)**	**α**	**β**	**Thr(V)**	**Δ(V)**	**PW(μs)**	**Current(μA)**
Fig2(D)(G)/Fig3(B)	800	100	15	0.937	1.499	1,200	0.01	−8.5	~	400	10:10:400
Fig2(C1)	800	100	15	0.907	1.499	1,200	0.01	−0.75	~	100	50:25:200
Fig2(C2)	800	100	15	0.907	1.499	1,200	0.01	−0.75	~	50:25:200	50
Fig2(E)	800	100	15	0.907	1.499	1,200	0.01	−0.75	~	50:25:200	5:5:150
Fig3(A)	800	100	15	0.907	1.499	1,200	0.01	−1.28	0.07	400	5:5:150
Fig3(D)	800	100	15	0.907	1.499	1,500	0.04	−1.28	~	400	2:5:150
Fig4(A1)	360	100	15	0.937	1.499	2,000	0.08	−5	~	5:5:1000	10:10:1000
Fig4(B1)	360	100	15	0.937	1.499	2,000	0.08	−5	~	150	10:10:1000
Fig4(A2)	240	100	8	0.937	1.499	2,000	0.08	−2.5	~	5:5:1000	10:10:1000
Fig4(B2)	240	100	8	0.937	1.499	2,000	0.08	−2.5	~	350	10:10:1000
Fig4(A3)	80	100	5	0.937	1.499	1,450	0.02	−4.6	~	5:5:1000	15:15:900
Fig4(B3)	80	100	5	0.937	1.499	1,450	0.02	−4.6	~	800	15:15:900
Fig5(A1)	100	100	15	0.937	1.499	1,200	0.01	−0.55	~	5:5:800	10:10:250
Fig6(1,1)	40	100	0.1	0.937	1.499	2,000	0.08	−5	~	5:5:1000	10:10:1000
Fig6(1,2)	40	100	8	0.937	1.499	2,000	0.08	−5	~	5:5:1000	10:10:1000
Fig6(1,3)	40	100	15	0.937	1.499	2,000	0.08	−5	~	5:5:1000	10:10:1000
Fig6(2,1)	80	100	0.1	0.937	1.499	2,000	0.08	−5	~	5:5:1000	10:10:1000
Fig6(2,2)	80	100	8	0.937	1.499	2,000	0.08	−5	~	5:5:1000	10:10:1000
Fig6(2,3)	80	100	15	0.937	1.499	2,000	0.08	−5	~	5:5:1000	10:10:1000
Fig6(3,1)	120	100	0.1	0.937	1.499	2,000	0.08	−5	~	5:5:1000	10:10:1000
Fig6(3,2)	120	100	8	0.937	1.499	2,000	0.08	−5	~	5:5:1000	10:10:1000
Fig6(3,3)	120	100	15	0.937	1.499	2,000	0.08	−5	~	5:5:1000	10:10:1000
Fig8(B&C)	500	100	10	1.2	0.55	1,200	0.01	−6	~	1000	100
Fig8(D1)	500	100	10	0.2	0.55	1,200	0.01	−6	~	5:5:1000	1:1:1000
Fig8(D2)	500	100	10	0.6	0.55	1,200	0.01	−6	~	5:5:1000	1:1:1000
Fig8(D3)	500	100	10	1.2	0.55	1,200	0.01	−6	~	5:5:1000	1:1:1000
Fig10(A1&A2)	120	100	0.1	0.937	1.499	2,000	0.08	−5	~	100	15:15:500

### 3.2. The moving track of the peaks

**Figure 5:**
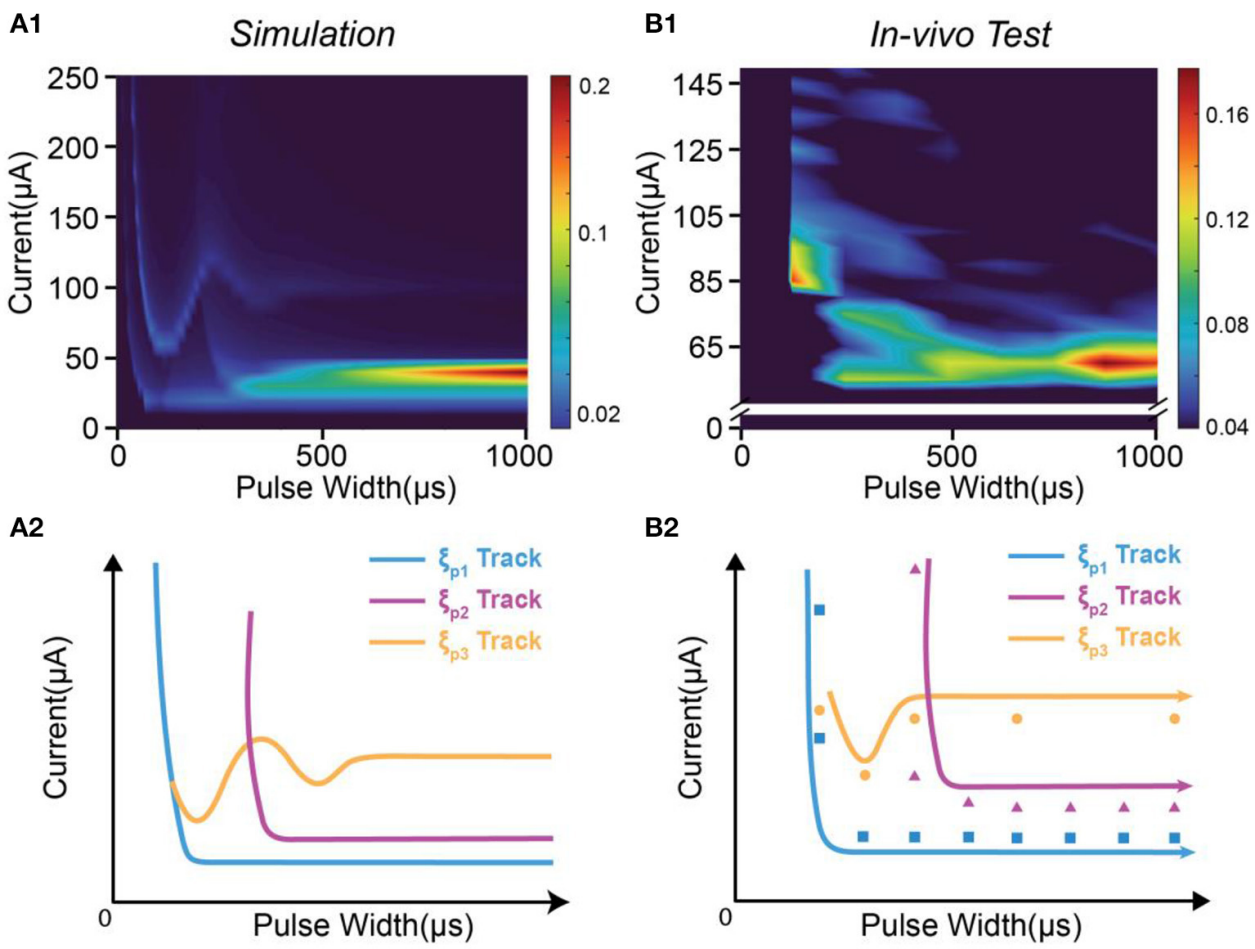
The moving tacks of instability peaks in *in-vivo* test are reproduced by modeling. **(A1)** The heat map of the instability by modeling; **(A2)** The tracks of the three instability peaks in **(A1)**; **(B1)** The measured heat map of the instability by *in-vivo* test; **(B2)** The recognized positions and tracks of the instability peaks in **(B1)**.

It is clearly observed that each peak moves along a specific track in the instability mapping. This moving track can also be reproduced by our modeling results shown in Figure 5. In Figure 5A1, we tune the modeling parameters to generate an instability mapping to fit the *in-vivo* results shown in Figure 5B1.

In Figure 5A1, three peaks can be found, named as ξ_*P*1_, ξ_*P*2_ and ξ_*P*3_. Their moving tracks are labeled in Figure 5A2. The moving tracks of ξ_*P*1_ and ξ_*P*2_ share a similar “L” shape. They have a large divergence at the low pulse width and tend to merge at the high pulse width. The moving track of ξ_*P*3_ has an individual shape with some oscillation.

The *in-vivo* results in Figure 5B1 can generally be fitted by the modeling results in Figure 5A1. The data to generate the heat map in Figure 5B1 is a sparse matrix, showing key features of the instability mapping. We labeled the position of the peaks of *in-vivo* testing in Figure 5B2. Based on the guidance of Figure 5A2, it is found that the positions of the peaks also closely fit the predicted moving tracks. The modeling parameters can be found in Table 2.

Since the modeling parameters mainly determine the instability mapping generated by C-P theory, we also generate some other possible patterns of the instability mapping by changing the circuit parameters. In this study, we only show how the patterns change with the quality factor, $Q=R_{P}\sqrt{\frac{C_{Membrane}}{L}}$, and the resistor connected in series with the inductor, *R*_*L*_.

**Figure 6:**
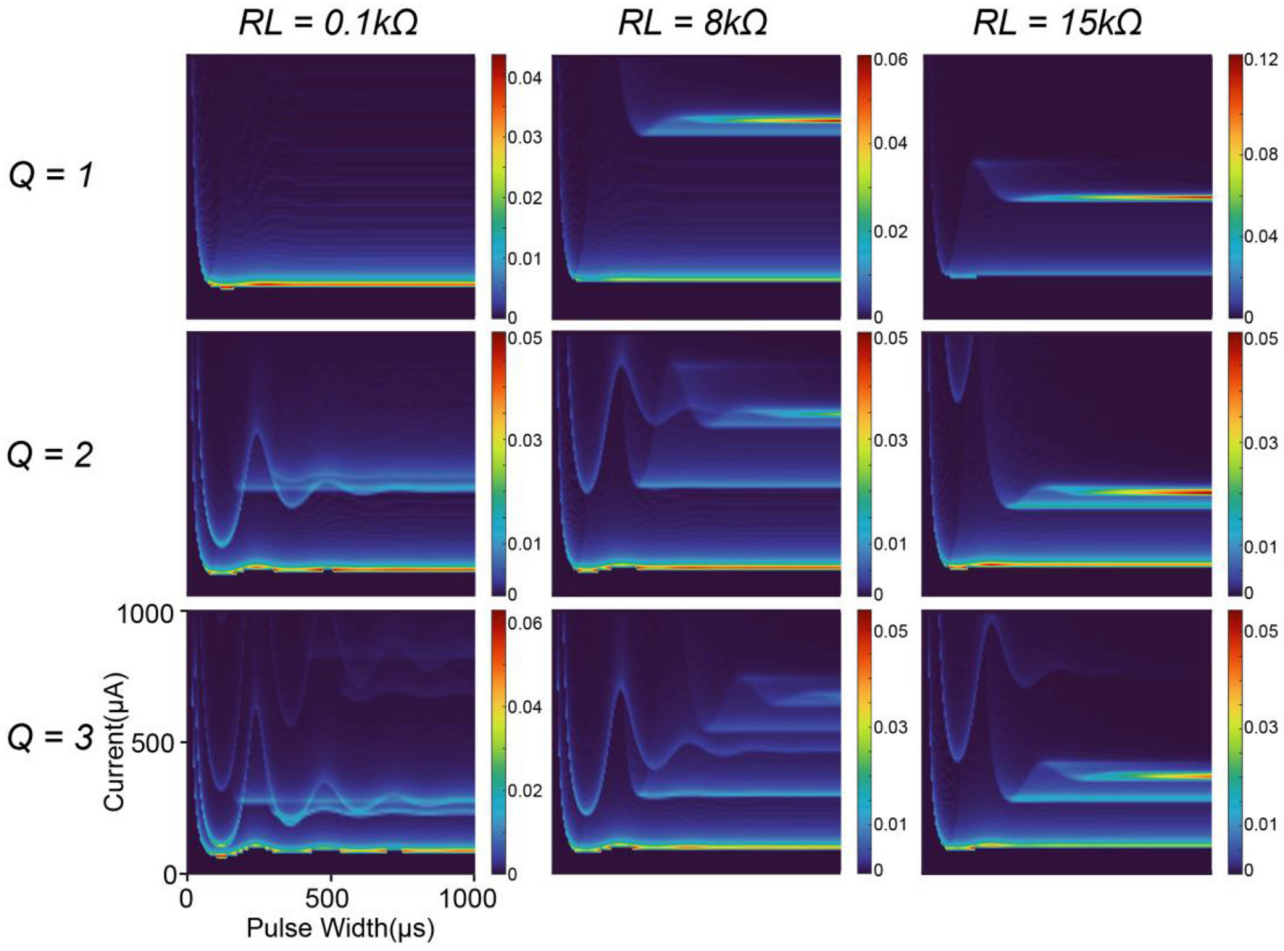
A parameter comparison by modeling. The resistor connected in series with the inductor and the quality factor of the circuit are selected as the variables.

In Figure 6, the quality factor, Q, mainly determines the spacing between the moving tracks of the peaks. A low Q factor will increase the spacing, while a high Q factor will make the tracks closer to each other. The resistor *R*_*L*_ mainly determines the density of the peaks. A lower *R*_*L*_ generates more peak tracks.

The patterns have quite complex changing trends, which cannot be fully elucidated here. Figure 6 only demonstrates a facet of the effect of the parameters on the modeling results, showing that the patterns and tracks of the peaks are highly tunable. The modeling parameters can be found in Table 2.

### 3.3. How to improve the ES-dependent stability by parameter selection

Our modeling work also provides a possible method to optimize the stimuli parameters to improve ES-dependent stability. We make a case demonstration based on the modeling results in Figure 5A1.

**Figure 7:**
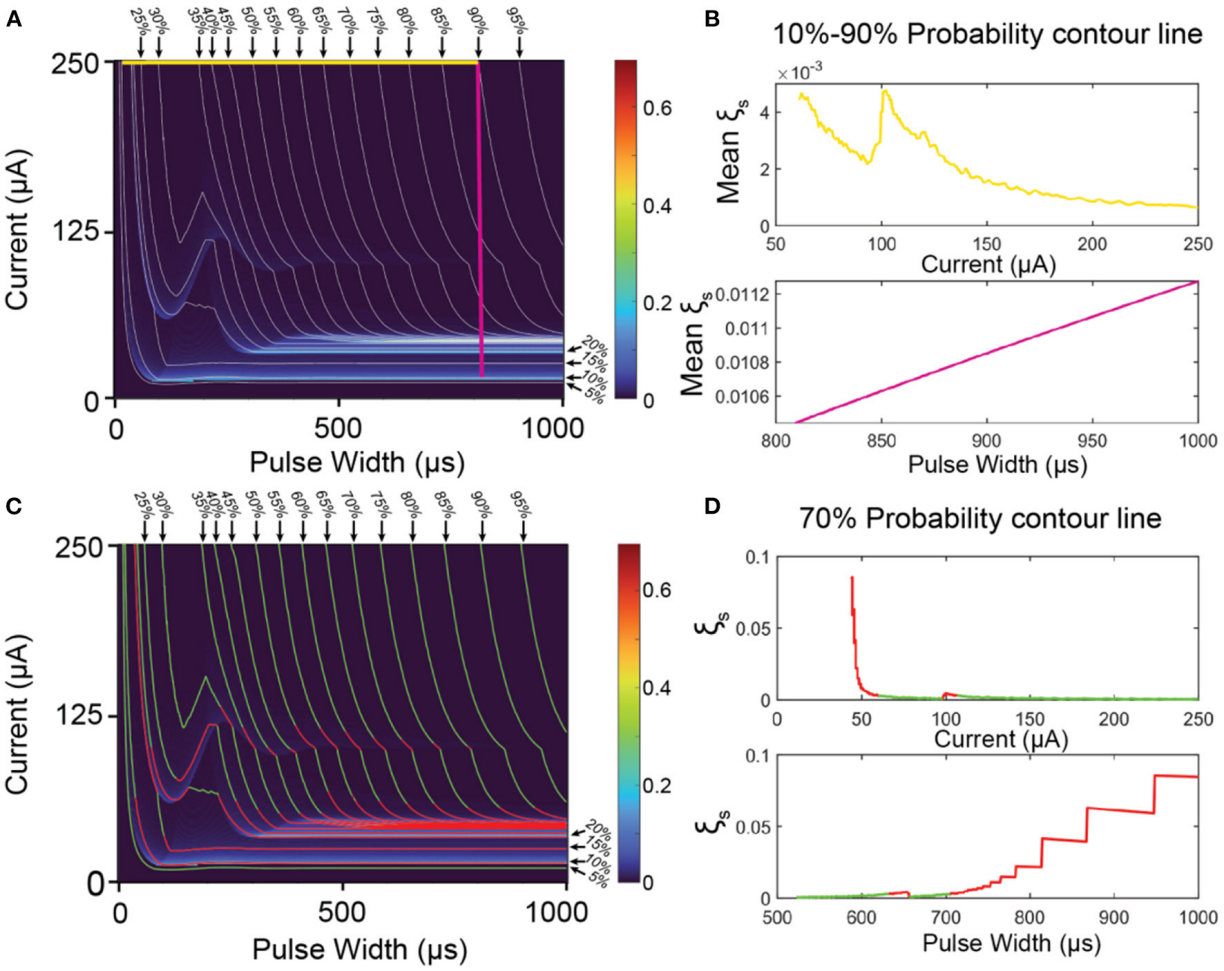
The methods to improve the ES-dependent stability by selecting proper stimuli parameters. **(A)** An overlapping of the contour map and its instability mapping. The horizontal yellow line is the parameter path with the minimal mean instability in all horizontal lines; the vertical purple line is the parameter path with the minimum mean instability in all vertical lines. **(B)** The calculated mean instability of all horizontal lines (yellow) and vertical lines (purple). **(C)** The same overlapping map as **(A)** with green region (low instability) and red region (high instability). **(D)** A case demonstration of the instability calculation of the 70% contour line by setting current (upper) and pulse width (lower) as x-axis. The curve higher than ξ_*Threshold*_ is labeled in red, while the curve lower than ξ_*Threshold*_ is labeled in green.

Since the C-P theory can generate the whole probability mapping for all stimuli parameters, we can also generate a contour map of the probability, as the gray lines shown in Figures 7A, C. Improving stimuli parameters is to find a path from a very weak stimulation (10%) to a very strong stimulation (90%) on the map (Figure 7A) with the lowest mean instability.

Firstly, we demonstrate the improving method by a simple parameter selection, which is fixing either the current amplitude or the pulse width. In this case, the path of the parameter in Figure 7A should be either a vertical straight line (fix the pulse width) or a horizontal straight line (fix the current amplitude). Averaging all the data points of the instability on each line for comparison, and the results are shown in Figure 7B. For the situation of fixing the current, the calculation results are shown as the yellow line in Figure 7B. This data curve shows some fluctuation and reaches the minimum at the current of 250 μA with mean instability of about 0.001, which refers to the yellow horizontal line at the top of Figure 7A. For the situation of fixing the pulse width, the results are shown as the purple line in Figure 7B. It is a monotonous increasing line with the pulse width. Thus, the minimal instability happens at about 810 μs with mean instability of about 0.0104, which refers to the purple line in Figure 7A. As seen, the instability of the purple line is about 10 times higher than that of the yellow line. The reason is that the vertical purple line inevitably goes through the region with very high instability (indicated with red and yellow colors, named as high instability region) at the bottom of Figure 7A, while the yellow line is located at the top of Figure 7A, which is a region with low instability. As seen, the key to improving the stability is to avoid the high instability region. Meanwhile, the modeling results in Figure 6 show that the high instability regions normally distribute horizontally. Thus, horizontal lines have a better chance of completely avoiding these regions. On the contrary, if the horizontal line accidentally across one of the high instability regions (which may happen in applications), all stimulations will exhibit high instability. Thus, it is recommended that a proper instability mapping, as in Figure 5A1, should be characterized to indicate the high instability regions. Just by avoiding these regions, ES-dependent stability can be significantly improved.

Apparently, if the parameter path is not a straight line, or even not a continuous path, it is possible to avoid all high-instability regions. To demonstrate this method, the instability of along each contour line is calculated. As a case demonstration, the calculation results of the contour line of 70% are shown in Figure 7D by setting pulse width and current as the x-axis, respectively. As seen, even with the same probability, the instability can have a dramatic difference by changing the stimuli parameters. Here we give a rule for setting the threshold for stimuli parameter selection:


$$\begin{array}{l} \xi_{Threshold=}\xi_{\min+}(\xi_{\max}-\xi_{\min})\;\times \;\;5\%; \end{array}$$

Here ξ_max_ and ξ_min_ refers to the maximum and minimum instability on the map, respectively. The region higher than ξ_*Threshold*_ is indicated with red color, while the region lower than ξ_*Threshold*_ is indicated with green color. As seen in Figure 7C, the green regions on the contour lines can roughly form the area, which parameter path can go through with very low instability.

This method may be challenging to apply in applications since a fine-characterized instability mapping is very difficult and time-consuming. The key inspiration of this modeling is that, even with the same probability, which refers to the stimulation strength, the instability can vary a lot by setting different parameters. Therefore, a proper parameter selection can significantly improve ES-dependent stability.

## 4. Discussion

### 4.1. The effect of the inductor involved in the equivalent circuit of neural tissue

**Figure 8:**
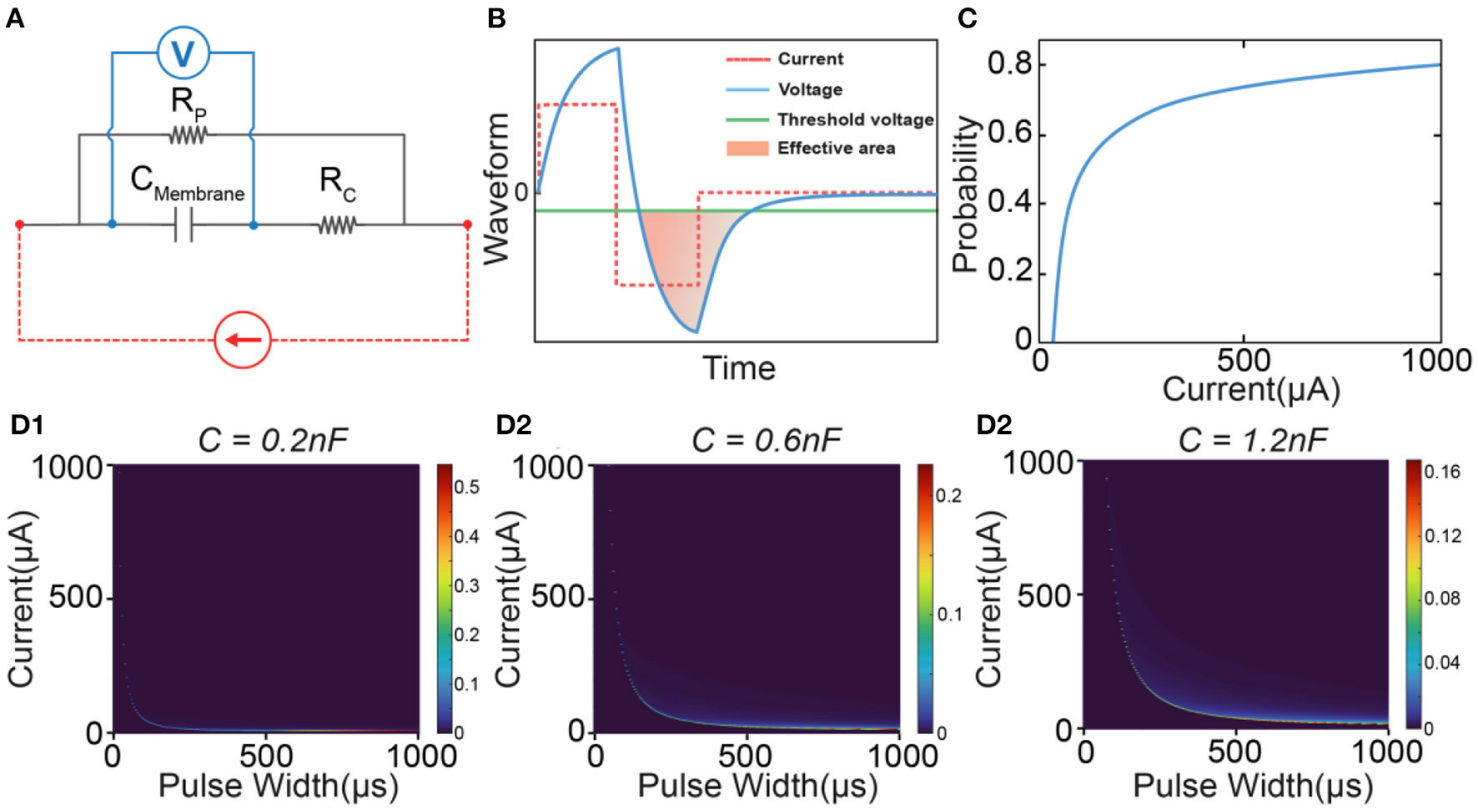
A comparison of the modeling by using an RC circuit. **(A)** The RC circuit used in modeling; **(B)** A representative voltage waveform (blue line) by applying a square current (red dash line). Only the voltage (filled with orange color) exceeding the threshold (green line) is calculated in probability calculus. **(C)** The probability is calculated by changing the current amplitude; **(D1–D3)** The heat map of the instability by changing the value of the capacitance.

The most controversial part of the C-P theory is the inductive factor involved in the equivalent neural circuit. Starting from the cable theory and H-H model, a neural circuit of RC configuration is always applied to model the axon. However, the passive voltage response does follow an RLC circuit, which has been validated by our previous works (Wang et al., 2020, 2021) and many previous studies (Sjodin and Mullins, 1958; Araki et al., 1961; Freeman, 1961; Ranck, 1963; Guttman, 1969; Mauro et al., 1970; Scott, 1971; Takashima and Schwan, 1974; Hombl and Jenard, 1984; Koch, 1984; Hutcheon and Yarom, 2000; Dwyer et al., 2012; Mosgaard et al., 2015). To further confirm the effect of the inductor on ES-dependent instability, the modeling results using an RC circuit (Figure 8A) are shown to make a comparison. A typical RC voltage response by applying a square current pulse is shown in Figure 8B. Due to the lack of oscillation, only one voltage block can exceed the threshold. Thus, the calculated probability shows a smooth increasing curve without any abrupt change (Figure 8C). Therefore, only one instability peak will be observed in modeling results (Figures 8D1–D3). The track of the instability peak will form an “L” shape, which is not affected by modeling parameters.

So based on our theory, if the neural circuit really follows an RC configuration, there shall always be only one peak, which is inconsistent with the experimental data. Meanwhile, compared with the simple peak track in Figure 8D, the instability mapping in Figure 5B1 shows a much more complex pattern of the tracks. Therefore, an RLC circuit can reproduce the passive property more accurately than an RC circuit.

### 4.2. The effect of current waveforms

**Figure 9:**
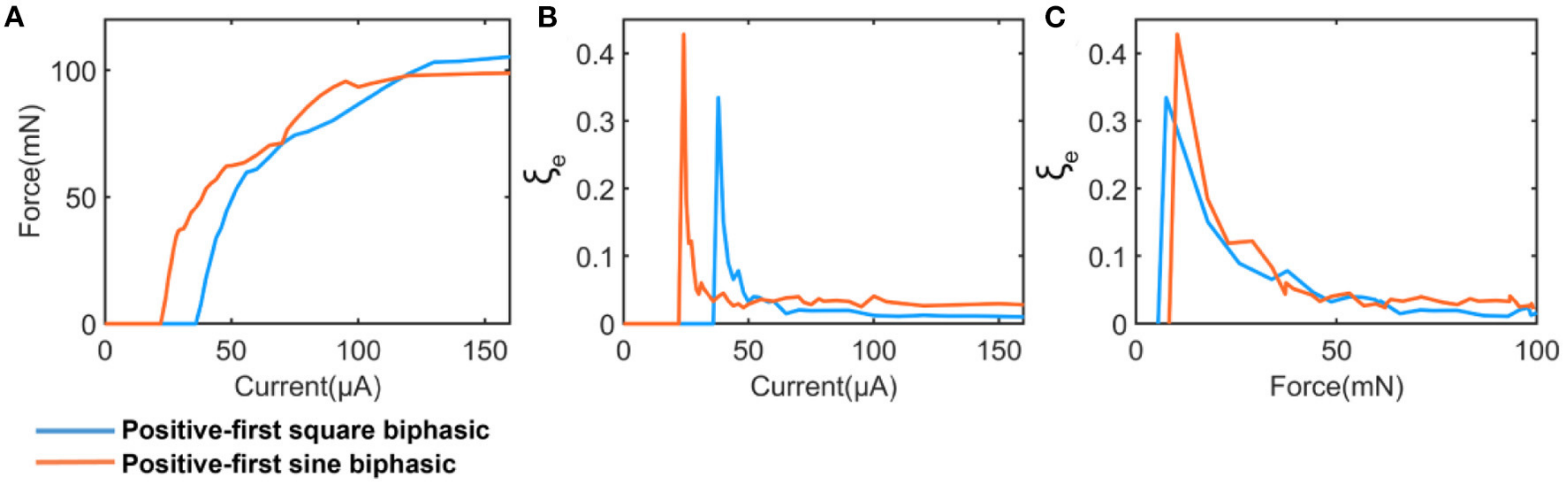
A comparison between square and sine current waveform. **(A)** The measured force; **(B)** The calculated instability curve; **(C)** Re-processed instability curves by setting the force as x-axis.

Our model suggested that the instability is also affected by the shape of the current waveform. To demonstrate this, two current waveforms, square wave and sine wave, were involved in the test shown in Figure 9. Figure 9A shows the measured force by changing the current amplitude. The applied pulse width is 200 μs. The calculated instability is shown in Figure 9B. We set the force as the x-axis, which provides a fair basis for the comparison (Figure 9C). Our results indicated that the sine wave induced a higher instability. It is worth investigating more with a wide range of stimulation waveforms and parameters in future studies.

### 4.3. Instability vs. linearity

It is expected to minimize the instability of the electrical stimulation. Meanwhile, it is also expected to achieve a linear control of the stimulation strength. However, based on our theory, there is a trade-off between these two factors. Based on the definition of instability, $\xi_{s}=\frac{P^{\prime }\left(I\right)}{P\left(I\right)}\;$, it can be derived that the ξ_*s*_ is directly proportional to *P*′(*I*), $P^{\prime }(I),\;\xi_{s}\propto \;P^{\prime }(I)$, while the linearity is directly proportional to *P*′(*I*), *Linearity***∝** *P*′(*I*). Thus, the instability ξ_*s*_ is directly proportional to the linearity, ξ_*s*_**∝** *Linearity*. It means the instability tends to be high in the range of electrical stimulation, which can provide a good linear relationship between the current amplitude and the neural response. While in the range that the strength of the neural response is not much affected by the current amplitude, the stimulation will be more stable. Thus, there is a trade-off between stability and stimulus settings.

**Figure 10:**
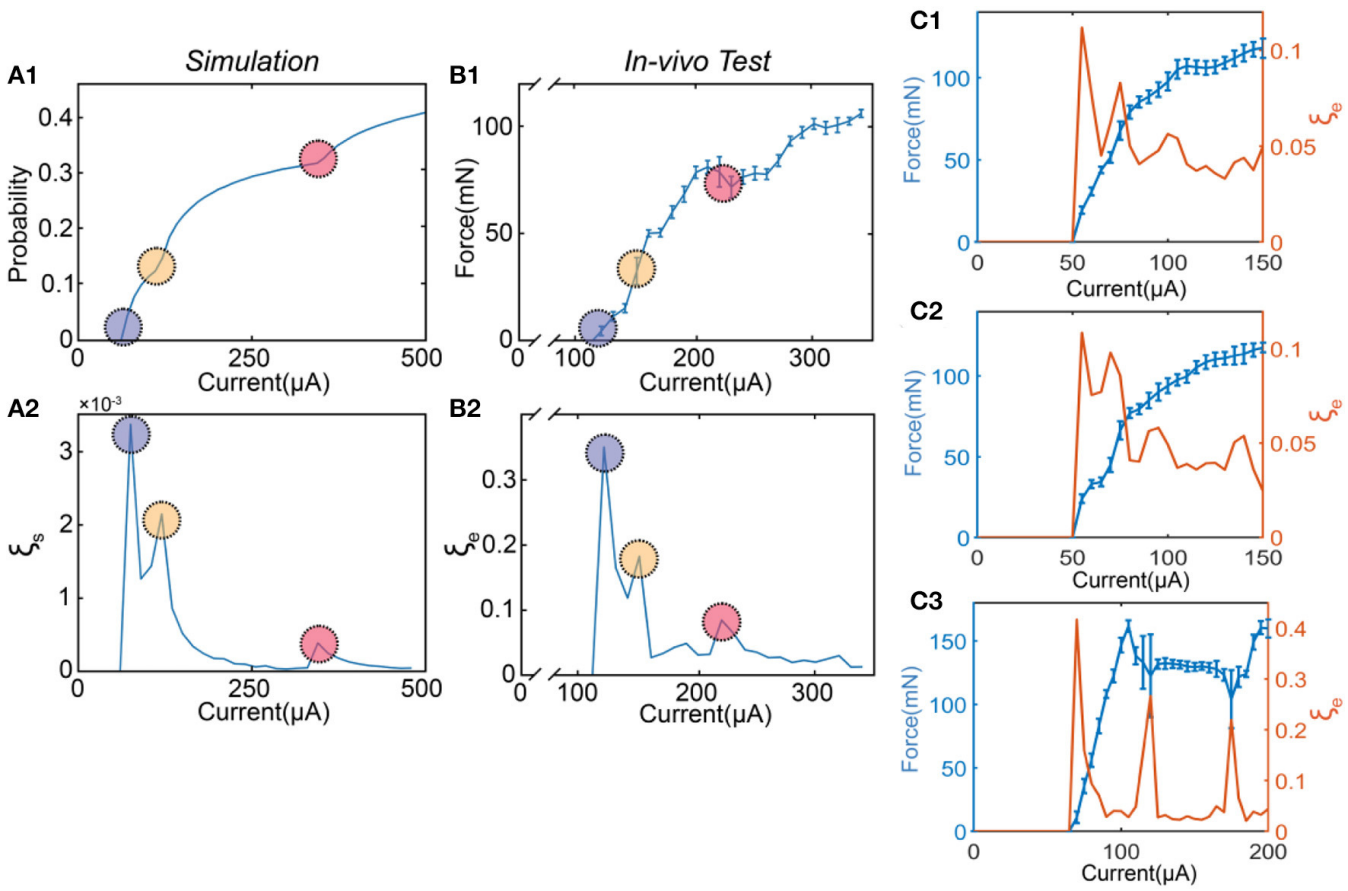
The modeling and *in-vivo* results show the trade-off between ES-dependent stability and linearity. **(A1)** A representative probability curve of modeling showing several abrupt change points; **(A2)** The calculated instability curve shows the positions of the peaks corresponding to the positions of the abrupt change points; **(B1)** A presentative force curve of the *in-vivo* test showing several abrupt change points; **(B2)** The instability curve shows the positions of the peaks corresponding to the positions of the abrupt change points. **(C1–C3**) The force-current curve with error bar (blue) and the instability-current curve (orange) shows other results of square wave tests on the sciatic nerve.

A more illustrative explanation of this trade-off is shown in Figure 10. Figure 10A1 shows the probability curve by changing the current amplitude, which can generally duplicate the testing results in Figure 10B1, the measured force by changing the current amplitude. The model parameters can be found in Table 2. In Figure 10A1, the probability curve is not smooth. Besides the beginning section, there are another two positions with abrupt changes labeled as circles with different colors. The derivatives of the probability curve at the circles reach the local maximum, corresponding to the three peaks in the instability curve (Figure 10A2). In Figure 10B1, similar abrupt change points can also be found. The instability curve in Figure 10B2 suggests that the peaks happen at the position of these abrupt changes. More similar data of *in-vivo* testing are shown in Figure 10C.

This study first proposed and validated the trade-off between stability and linearity in electrical stimulation. The prediction and explanation of this trade-off show that our theory does not only fit the testing data but also elucidates its mechanism.

### 4.4. The issue of historical path divergence

**Figure 11:**
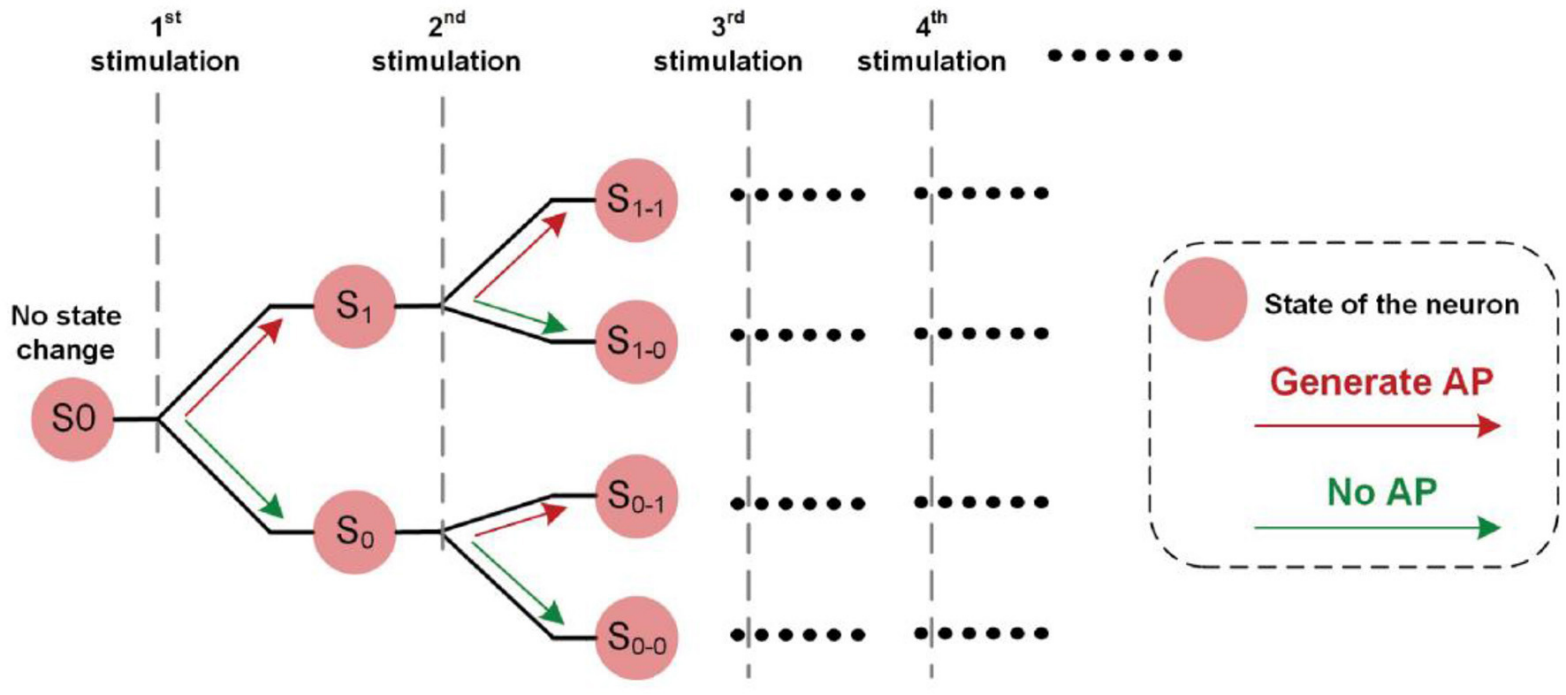
The illustrative explanation of the historical path divergence issue.

We proposed a new concept called historical path divergence. It is essential to understand the phenomenon of continuous electrical nerve stimulation. A brief illustration of this concept is shown in Figure 11.

In Figure 1E3, the fluctuation of the measured force demonstrated a non-monotonic trend. So, it can be inferred that the state change of the neuron, which refers to the threshold fluctuation, is determined by both the electrical input and the real-time neuron states.

At the beginning of the electrical stimulation, all neurons are at state S0, which means no state change. Since all neurons are in the same state, they are synchronized. After the first stimulation is applied, two possible outcomes might occur: S_1_ (generate AP) and S_0_ (no AP). Since the number of state combinations for all neurons increases, they are less synchronized or more asynchronous (that is, neurons tend to have different states).

Each state can have two possible outcomes when the subsequent stimulation is applied. Therefore, the number of possible states is increased, causing decreased synchronicity of neurons.

The stimulus-induced historical paths formed a binary tree in Figure 11. The synchronicity of the states of all neurons will determine the observed instability. When the synchronicity is higher, such as state S0, all neurons tend to have the same state change post-stimulus. This state change can either increase or decrease excitability. Therefore, the observed force will have a more evident fluctuation trend, either increasing or decreasing. With subsequent stimulations, neurons tend to be asynchronous. Some may increase, and some may decrease. Thus, the observed force fluctuation will not have an evident changing trend. In other words, the measured force can be more stable.

At the beginning of the stimulation, the synchronicity across neurons is high. Thus, the resulting force tends to have a more evident changing trend with more increased instability. Meanwhile, this evident changing trend is the same in all testing trials. However, with subsequent stimuli, this evident changing pattern tends to disappear, and the force tends to be more stable.

**Figure 12:**
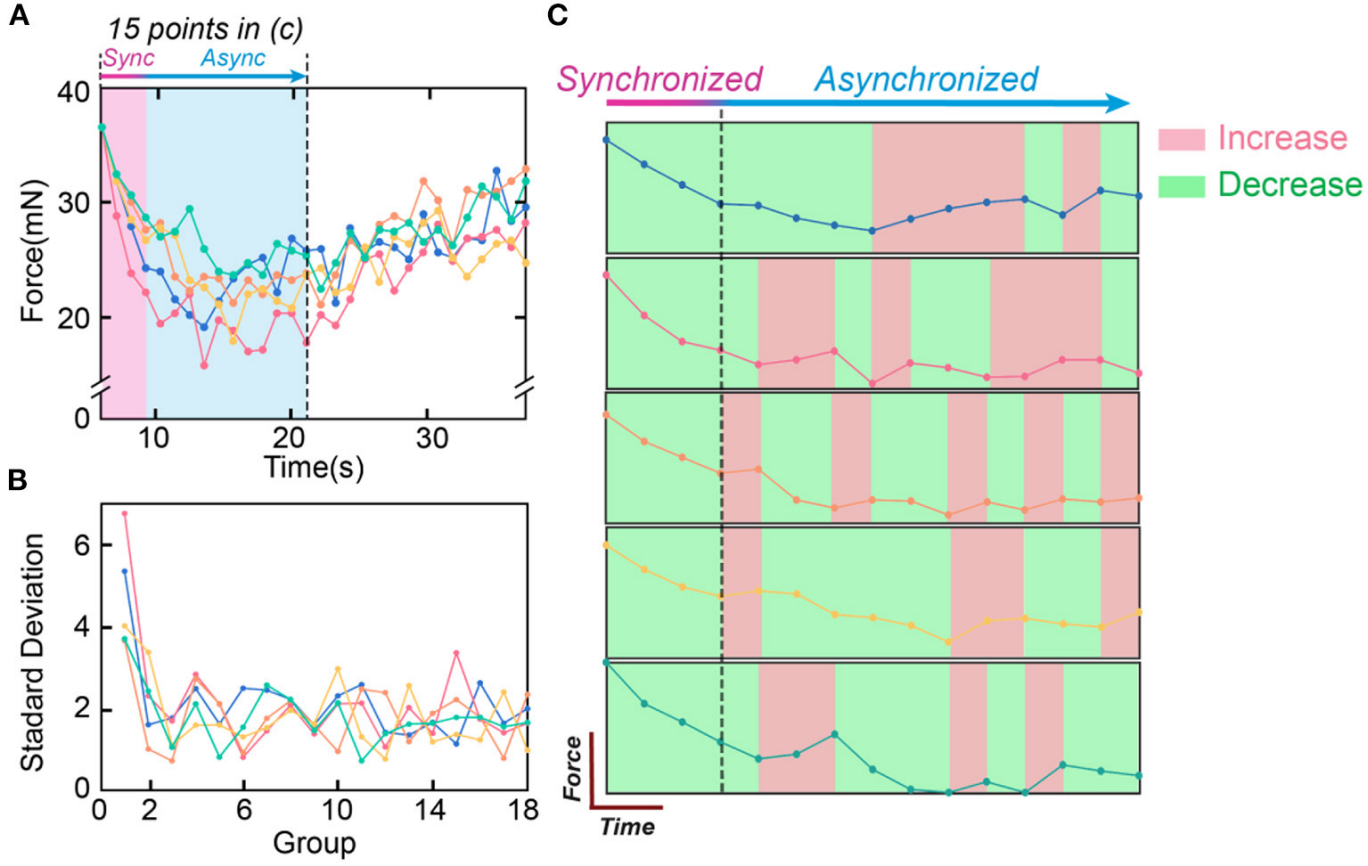
The testing data to validate the historical path divergence issue. **(A)** The five measured force curves with the same testing parameters, and the synchroized and asynchronized area in **(C)**; **(B)** The standard deviation of the force curve in **(A)**. Every five points in **(A)** are calculated as one data point in **(B)**; **(C)** The first fifteen points of the five curves in **(A)** are compared. The changing trends are labeled in colors (red refers to increasing, and green refers to decreasing).

The abovementioned conclusions are validated by the *in-vivo* experiments shown in Figure 12. Figure 12A shows the curves of five testing trials with the same stimulation parameters. The standard deviation (SD) can represent the instability of the curve. To show the change in the SD with time, the SD is calculated with each 5 data points (Figure 12B). SD is high at the beginning and soon decreases to a certain level, showing the trend that the force becomes more stable with time. Figure 12C only shows the first 15 data points of the force curve in Figure 12A. The changing trend is labeled with colors (green refers to decrease, and red refers to increase). Only at the beginning stage (the first 4 data points), all force curves show the same decreasing trend.

The data analysis in Figure 12 shows the effect of the historical path divergence. Continuous stimulation will diverge the historical paths of all neurons and reduce instability. Thus, instability is always the highest at the beginning stage.

## 5. Limitations

Although we can reproduce the patterns of peaks in the modeling results, the heights of the peaks are not accurate. Several factors might limit the modeling accuracy.

### 5.1. The parameters in the C-P theory are not accurate

We cannot precisely assign the parameters in C-P theory. Our previous work developed the C-P theory to build the mathematical relation between the electrical input and the neural response, which is similar to the H-H model. However, unlike the H-H model, the C-P theory can describe macroscopic electrical nerve stimulation. Thus, those parameters involved in the model are not measurable. Meanwhile, due to the high nonlinearity of the model, it is also difficult to precisely estimate these parameters. Currently, we assign the value of each parameter by the exhaustive method, which can roughly reproduce the distinctive features of the testing results, such as the number and position of the peaks.

### 5.2. The value of threshold fluctuation is unknown

The instability's origin is known as the membrane potential fluctuation. However, it is difficult, if not impossible, to measure the value and range of the membrane potential fluctuation *in-vivo*. This value will quantitatively affect the height of the peaks in our modeling results but does not affect the general patterns. Therefore, we give a new definition of instability free of the value of membrane potential fluctuation, $\frac{P^{\prime }}{P}$, which is more suitable for qualitatively reproducing the patterns of the peaks.

### 5.3. The definitions of instability of *in-vivo* testing and modeling are not consistent

The instability of *in-vivo* testing is based on the measured force's standard deviation (SD). However, the SD cannot be generated in our modeling. Instead, we used the difference between the maximum and minimum probability, which positively correlated with the SD. This inconsistency will not affect the reproduction of the patterns but will forbid us from acquiring the accurate height of peaks.

## 6. Conclusion

There is much-growing evidence that the performance of electrical stimulation cannot be significantly improved by merely optimizing stimulus parameters without considering the complexity of the biophysical characteristics of the target nerve system. Under certain specific stimuli parameters, the ES-dependent instability can reach a local maximum. We investigated the characteristics of ES-dependent instability by the Circuit-Probability theory. Our model reveals several critical characteristics of the instability peaks. Firstly, the instability peaks' physical origin is the axon's oscillatory nature, whose passive electric property shall be modeled by an RLC circuit. Thus, due to the complexity of the RLC circuit's voltage response, the measured instability peaks will follow certain patterns which our model can reproduce. On this basis, ES-dependent stability can be improved by selecting appropriate parameter paths to bypass the instability peaks. We demonstrated different methods to search optimal parameter paths, either a continuous straight path or a discontinuous arbitrary path, to minimize the total instability of traversing all probability levels. Meanwhile, in our model, the measured pattern of instability peaks of *in-vivo* testing is derived from the passive response of neural circuits. It substantially supports the necessity of adding an inductive factor in neural circuits. Moreover, our model reveals the trade-off between ES-dependent stability and linearity. That is, given a current range that allows linear control of stimulation strength, the stability of neural response tends to be low. Finally, our model proposes a new perspective to investigate electrical nerve stimulation: the historical path divergence that predicts ES-dependent instability changes with the stimulation duration, which means that the instability is always the highest in the initial stage of stimulation, and gradually decreases with the increase of stimulation duration.

## Data availability statement

The original contributions presented in the study are included in the article/supplementary material, further inquiries can be directed to the corresponding authors.

## Ethics statement

The animal study was reviewed and approved by Shenzhen Institutes of Advanced Technology, Chinese Academy of Sciences (SIAT-IACUC-210623-YGS-WH-A1965).

## Author contributions

HW proposed the theory. SY carried out the modeling process. SY, YL, WY, and YZ carried out the *in-vivo* tests. SK and WY fabricated the neural probes. TZ, ZX, and FL helped train the experimental process. TG helped refine the theory and improve the writing. BS, TW, and XY provided the general guidance of the study. All authors contributed to the reference collection, idea discussion, contributed to the article, and approved the submitted version.
